# Mitogenomic and Metabarcoding Resources for the Study and Conservation of Keystone Neotropical Raptors

**DOI:** 10.1002/ece3.73262

**Published:** 2026-03-17

**Authors:** Diego De Panis, Octavio Priotto, Julián Padró

**Affiliations:** ^1^ Leibniz Institute for Zoo and Wildlife Research Berlin Germany; ^2^ Grupo de Investigaciones en Biología de la Conservación, INIBIOMA, Universidad Nacional del Comahue—CONICET Bariloche Argentina

**Keywords:** diurnal raptors, genetic monitoring, metagenomics, phylogentic markers

## Abstract

Neotropical raptors are among the most threatened birds, facing increasing extinction risks due to habitat loss and human persecution. Despite their importance for ecosystem stability, basic data on their distribution, abundance, and genetic diversity remain scarce. To address these gaps, we assembled and annotated the mitochondrial genomes of nine high‐priority raptors from the Neotropics, including the threatened Chaco Eagle (*Buteogallus coronatus*), Black‐and‐Chestnut Eagle ( *Spizaetus isidori* ), Rufous‐tailed Hawk ( *Buteo ventralis* ), and Harpy Eagle ( *Harpia harpyja* ), as well as the Near Threatened Orange‐breasted Falcon ( *Falco deiroleucus* ), Crested Eagle ( *Morphnus guianensis* ), Ornate Hawk‐Eagle ( *Spizaetus ornatus* ), Plumbeous Hawk (*Cryptoleucopteryx plumbea*), and Solitary Eagle (*Buteogallus solitarius*). Mitogenome sizes ranged from 17,848 to 20,449 bp, with consistent gene content and a Control Region architecture common in Falconidae and Accipitridae. Phylogenetic analyses provided strong support for most relationships, highlighting the value of mitogenomic data for phylogeographic studies. We further designed metabarcoding primers for environmental DNA applications. Primers targeting the 12S rRNA gene and a mini‐barcode for the Harpy Eagle's Control Region showed high resolution using short, conserved sequences ideal for combining degraded DNA with next‐generation sequencing. Our study provides essential molecular tools for monitoring and protecting these ecologically vital yet threatened raptors across the Americas.

## Graphical abstract

We characterized complete mitochondrial genomes for nine keystone Neotropical raptors, including four threatened and five near‐threatened species, providing essential resources for phylogenetic and conservation studies. We also developed metabarcoding primers, enabling non‐invasive monitoring of Neotropical raptors via environmental DNA. These genetic tools support biodiversity assessments, population monitoring, and conservation planning for elusive and threatened raptors across the Americas. Graphical photo abstract: *Spizaetus isidori* from Ecuador (photo: Jaime Culebras).

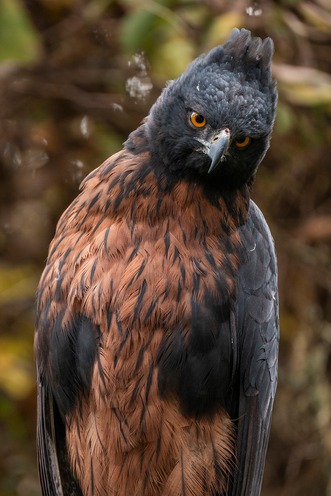

## 1 Introduction

**FIGURE 1:**
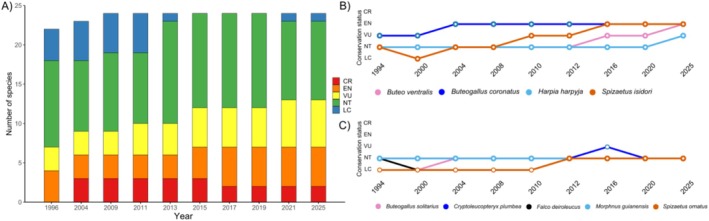
Changes in IUCN conservation status of 24 Neotropical raptor species of conservation concern across years (A). Trajectories of the four threatened species (B) and five near‐threatened species (C) included in this study (CR = Critically Endangered, EN = Endangered, LC = Least Concern, NT = Near Threatened, VU = Vulnerable).

Neotropical raptors play a critical ecological role across the diverse landscapes of southern North America, Central America, and South America. The region harbors nearly one‐third of all diurnal raptors known so far (97 species comprising three orders: Accipitriformes, Falconiformes, and Cathartiformes; Sarasola et al. 2018). Nearly a quarter of all Neotropical raptor species are of conservation concern (two Critically Endangered, five Endangered, five Vulnerable, and eleven Near Threatened; Figure 1A, Table S1), primarily due to persecution, contaminants, and habitat destruction (BirdLife International 2025). Despite recent calls for increased and standardized monitoring efforts (Perrig et al. 2019; Gousy‐Leblanc et al. 2021; McClure et al. 2023, 2025), information on genetic diversity, distribution, abundance, and population trends remains scarce (McClure et al. 2018; Buechley et al. 2019). To help address this gap, we focus on a set of threatened and near‐threatened Neotropical raptors for which genetic tools are lacking (Figure 1B,C).

The endangered Chaco Eagle (*Buteogallus coronatus*) is one of South America's rarest eagles, inhabiting semi‐open landscapes of Brazil, Bolivia, Paraguay, and Argentina, with fewer than 2000 mature individuals remaining and major threats from habitat loss, persecution, and electrocution (Do 2020; Sarasola et al. 2020; BirdLife International 2024a). The Black‐and‐chestnut Eagle ( *Spizaetus isidori* ), restricted to Andean forests, is similarly endangered, with an estimated 1400–4200 mature individuals and continued declines driven by deforestation and human conflict (Zuluaga et al. 2022; BirdLife International 2024b; Rivas‐Fuenzalida, Grande, et al. 2024; Restrepo‐Cardona et al. 2024). The Rufous‐tailed Hawk ( *Buteo ventralis* ), confined to temperate forests of southern Chile and Argentina, was recently reclassified as endangered following population estimates of 700–3300 individuals, threatened by logging and human persecution, yet its ecology and population structure remain poorly understood (Rivas‐Fuenzalida, Burgos‐Andrade, et al. 2024; BirdLife International 2025). The Harpy Eagle ( *Harpia harpyja* ), although more abundant (100,000–250,000 mature individuals) and widespread, ranging from southern Mexico to northern Argentina, occurs patchily across its range and is classified as Vulnerable due to deforestation and persecution. Nevertheless, its true status remains uncertain in many regions (Schulenberg 2020; BirdLife International 2021).

Among the near‐threatened species are five forest‐associated raptors that occur at low densities and remain poorly documented across much of their range. The Orange‐breasted Falcon ( *Falco deiroleucus* ) occurs primarily at a limited number of nesting sites across its former range from southern Mexico to northern Argentina, with an estimated 5000–25,000 mature individuals threatened, primarily by deforestation (Berry et al. 2020). The Solitary Eagle (*Buteogallus solitarius*) is a rare large buteonine with a patchy distribution from western Mexico to northwest Argentina, and an estimated population of 1000–2499 mature individuals, threatened mainly by forest loss and persecution (Phillips and Clark 2023). The Plumbeous Hawk (*Cryptoleucopteryx plumbea*) is among the least known Neotropical raptors. Mainly restricted to the Pacific coast of Colombia and Ecuador, its population is estimated at 10,000–19,999 mature individuals, likely declining due to ongoing deforestation (Bierregaard et al. 2020; BirdLife International 2020). The Ornate Hawk‐Eagle ( *Spizaetus ornatus* ) and the Crested Eagle ( *Morphnus guianensis* ) are wide‐ranging forest eagles occurring at low densities, from northern Central America to the Amazon Basin and the Atlantic Forest. Both species require extensive territories of mature forest, but have limited demographic information available and face ongoing population declines driven primarily by habitat loss, fragmentation, and persecution (Iliff 2020; Smith 2020).

These raptor species share key traits, such as low population densities, large home ranges, and fragmented distributions in dense forest habitats that severely limit detectability using traditional survey methods, thereby hindering effective assessments of their true conservation status across regions (e.g., Anderson et al. 2015; Robinson et al. 2018). A novel approach to address these challenges is the use of environmental DNA (eDNA) to study wild populations without the need for direct observation (Deiner et al. 2017; Ruppert et al. 2019; Clare et al. 2022). Coupled with metabarcoding and next‐generation sequencing, eDNA allows detection of genetic material shed into the environment, enabling assessments of species presence, abundance, diversity, and connectivity (Nakajima and Tsuri 2024; Ai et al. 2025; Cramer et al. 2025). This approach can reduce reliance on invasive sampling and labor‐intensive field surveys, while complementing traditional monitoring methods (Padró 2024). However, its effectiveness depends on the availability of comprehensive genetic reference databases, and commonly used mitochondrial markers (e.g., COI, CytB, 12S) remain scarce for many raptors, particularly those at high risk of extinction (Perrig et al. 2019; Gousy‐Leblanc et al. 2021), limiting both biodiversity monitoring and evolutionary research (Deiner et al. 2017; De Panis et al. 2021).

Herein, we provide the first annotated mitochondrial genomes for the four threatened and the five near‐threatened and data‐deficient Neotropical raptors. We further developed eDNA metabarcoding markers to improve species detection, resolve taxonomic uncertainty, and enhance phylogeographic and evolutionary analyses. By expanding the genetic toolkit available for conservation, we facilitate the transition toward genetic‐informed conservation programs and evolutionary research of these imperiled birds of prey.

## 2 Material and Methods

### 2.1 Assembly and Annotation

We assembled and annotated the complete mitochondrial genomes of the following keystone Neotropical raptors using whole‐genome sequencing (WGS) data from publicly available repositories: *B. coronatus* (formerly *Harpyhaliaetus*; SRR17853877), *H. harpyja* (SRR25728242/6/7), *B. ventralis* (SRR17454564), *F. deiroleucus* (SRR24451039), *B. solitarius* (SRR17835758), *C. plumbea* (SRR19616483), *S. ornatus* (SRR18186383) and *S. isidori* (SRR33778677‐80) WGS sequences (e.g., Catanach and Pirro 2023; Martin et al. 2024). In addition, we annotated the mitogenome of *M. guianensis* (bMorGui1.MT.20240212) assembled from the WGS data (GCA_045345515.1). For Illumina data, we used the assemblers NOVOPlasty v4.3.3 (*mito* assemble mode, *k‐mer = 23*, and expected genome size of 15–25 kb, using quality scores and automatic insert size with reduced ambiguous *N*'s) and GetOrganelle v1.7.5 (assemble type *‐F animal_mt*, with max extension rounds of *‐R 5*, using SPAdes algorithm and *k‐mer = 21*), selected for their efficiency in recovering circular mitogenomes from short‐read sequences (Dierckxsens et al. 2017; Jin et al. 2020). PacBio HiFi and Oxford Nanopore long reads were assembled with MitoHiFi v3.2.2 (using primary contig mode *‐‐primary* with frequency‐based read filtering disabled *‐f 0*) and Flye 2.9.6 (ONT reads were mapped against closely related mitogenomes < 25 kb using *‐nano‐hq* and final assembly was polished using *‐‐polish‐target*), leveraging highly contiguous sequences (Kolmogorov et al. 2020; Uliano‐Silva et al. 2023). Assembler algorithms were seeded with the longest available mitochondrial sequence per species, prioritizing the control region (CR) when present (Table S2). The resulting assemblies were annotated with MITOS v2 (Bernt et al. 2013) under the vertebrate mitochondrial genetic code (*‐c 2*), using Metazoa (*‐r RefSeq89f*) as a reference with default parameters. Annotations were visualized as organelle genome maps with OGDRAW v1.3.1 (Greiner et al. 2019). To confirm species identity and exclude potential contamination, we conducted in silico validation using BLASTn (Chen et al. 2015) on informative markers (e.g., COI, CR, CytB) against NCBI records and verified sequence identity against published references for each species (Table S2). All protein‐coding genes (PCGs), rRNAs, tRNAs, and the CR were manually curated.

### 2.2 Phylogenetic Analysis

To test the phylogenetic signal of our mitogenome dataset, we conducted comprehensive analyses incorporating 29 additional mitogenome sequences of diurnal raptor species (representing each genus) available from GenBank (Table S3). Our taxonomic sampling included a total of 38 raptor species representing Falconidae (5), Cathartidae (5), Sagittariidae (1), Pandionidae (1), and Accipitridae (26), plus nine outgroup taxa from Galliformes and Anseriformes. All analyses were performed using the PhyloSuite v1.2.3 bioinformatics pipeline (Zhang et al. 2020). We standardized mitogenome annotations and performed sequence alignments with MAFFT v7.526 (Rozewicki et al. 2019) employing the codon‐aware mode for PCGs and standard mode for RNAs. PCG alignments were subsequently refined with MACSE v2.04 (Ranwez et al. 2011), and ambiguously aligned regions were filtered with GBLOCKS v0.91b (Talavera and Castresana 2007) using conservative parameters (minimum block length = 10; gaps allowed). ModelFinder (Kalyaanamoorthy et al. 2017) was implemented to determine optimal partition schemes and substitution models through Bayesian Information Criterion scores, with unlinked branch lengths across partitions. We used the informative mitochondrial gene set (13 PCGs + 2 rRNAs) for the final phylogenetic reconstruction (De Panis et al. 2021), employing the maximum likelihood approach of IQ‐TREE v2.4.0 (Nguyen et al. 2015) with 10,000 Ultra Fast Bootstraps, with 1000 iterations and 1000 replicates under a partitioned model.

### 2.3 Metabarcode Assessment

To develop taxon‐specific metabarcoding primers for Neotropical raptor eDNA monitoring, we implemented bioinformatic analyses for in silico primer design (Ficetola et al. 2010). First, we compiled a reference database using BLASTn (Chen et al. 2015) to query our annotated COI, CytB, and 12S sequences (loci with established discriminatory power). We retained all Neotropical sympatric raptor species from GenBank to compile the target database, reaching a total of 135 sequences from 57 species (Table S2). These curated sequences were analyzed in the ecoPrimers package (Riaz et al. 2011) to develop potential barcode primers with taxonomic resolution for eDNA metagenomic applications. We used standard parameters: a maximum of three mismatches allowed, minimized amplicon lengths (prioritizing < 300 bp for degraded eDNA and NGS compatibility), and optimization for GC content and melting temperature uniformity. Candidate primers were further filtered to minimize dimer formation and maximize binding coverage (Bc), defined as the proportion of target taxa with perfect primer matches. Additionally, we used the ecoPCR package (Ficetola et al. 2010) to further validate in silico specificity against the reference database (Bellemain et al. 2010). We also applied this pipeline to all 22 known Harpy Eagle CR haplotypes (~400 bp; Table S2) to develop eDNA minibarcode primers for intraspecific resolution, enabling potential haplotype‐level population monitoring (Lerner et al. 2009). This approach prioritized primer sets that balance amplification efficiency with taxonomic discrimination, ensuring suitability for eDNA applications where template DNA is often fragmented and present in low quantities.

## 3 Results

**FIGURE 2:**
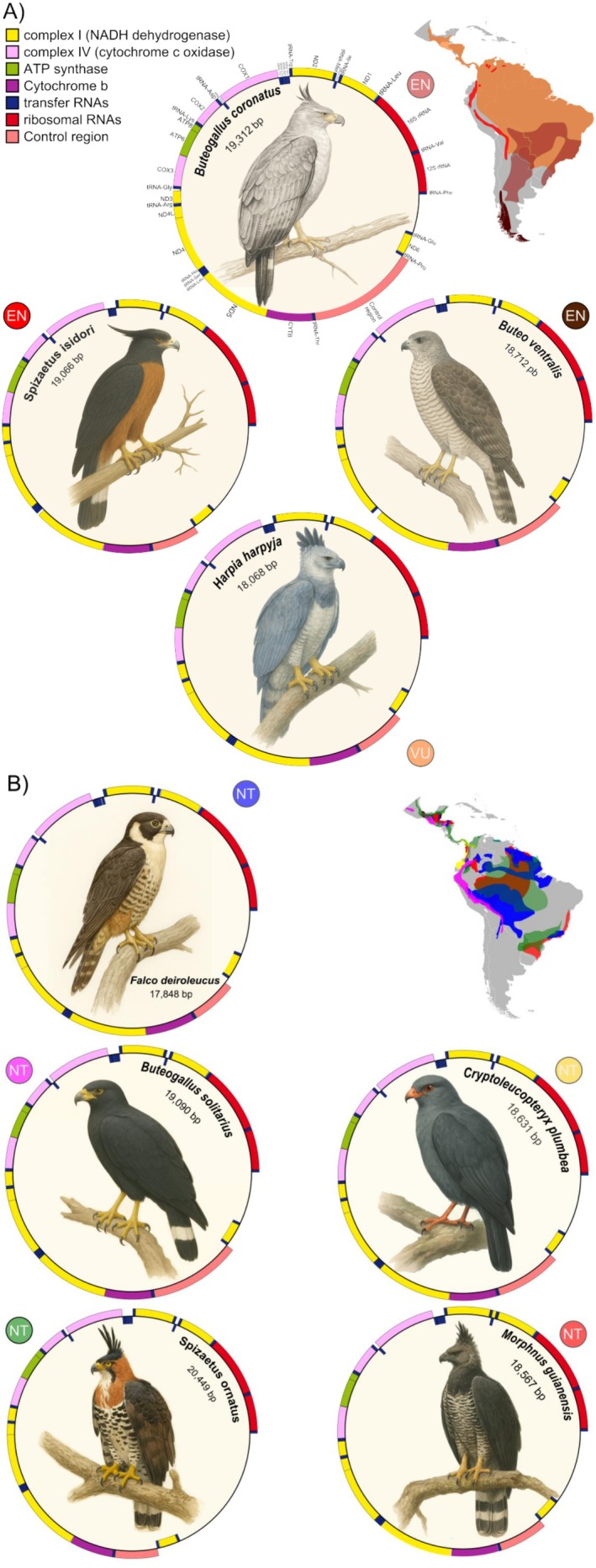
(A) Physical map of the complete mitochondrial genomes of four threatened top avian predators of the Neotropics. The conservation status of each species is indicated by colored circles, and their distributional ranges are shown on the map (color‐coded). (B) Physical map of the complete mitochondrial genomes of five near‐threatened top avian predators of the Neotropics. The conservation status of each species is indicated by colored circles, and their distributional ranges are shown on the map (color‐coded).

We assembled and annotated complete mitochondrial genomes for nine emblematic Neotropical raptor species, including four threatened taxa ( *B. coronatus* , *S. isidori* , *H. harpyja* , and *B. ventralis* ) and five near‐threatened and data‐deficient species ( *F. deiroleucus* , *B. solitarius* , *C. plumbea* , *S. ornatus* , and *M. guianensis* ). Mitochondrial genome sizes ranged from 17,848 to 20,449 bp, with *F. deiroleucus* presenting the smallest genome and *S. ornatus* the largest. All assemblies contained the full set of 37 mitochondrial genes typically found in vertebrates, including 13 protein‐coding genes (PCGs), 22 transfer RNAs (tRNAs), two ribosomal RNAs (rRNAs), and a Control Region (CR; Figure 2). Four genera were represented for the first time by complete mitochondrial genomes. Additionally, several commonly used identification markers were newly characterized for multiple species, including COI and 12S for six species, the Control Region for five species, and CytB for one species (Table S2). Overall nucleotide composition was consistent across species (A ≈ 31.0%, C ≈ 31.2%, G ≈ 13.9%, T ≈ 23.9%), showing a slight A + T bias (Figure S1). The Control Region length varied from 1046 bp in *F. deiroleucus* to 2777 bp in *B. coronatus* . In all species, the Control Region was located between tRNA‐Thr and tRNA‐Pro. In addition, a variable pseudo‐Control Region was detected between tRNA‐Glu and tRNA‐Phe (Figure 2).

**FIGURE 3:**
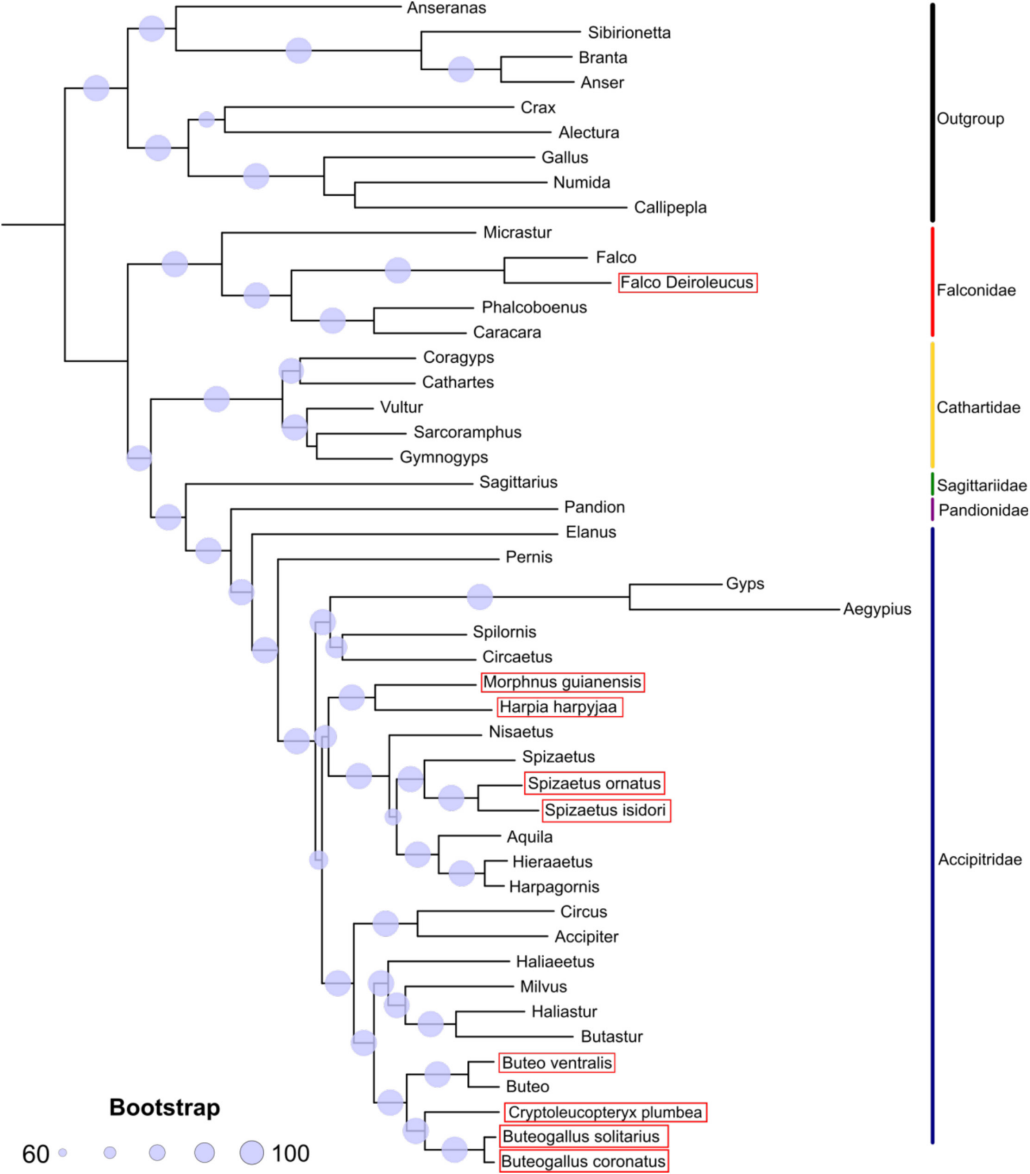
Phylogenetic relationships of the Orange‐breasted falcon ( *F. deiroleucus* ) Crested Eagle ( *M. guianensis* ), Harpy Eagle ( *H. harpyja* ), Ornate Hawk‐Eagle ( *S. ornatus* ), Black‐and‐Chestnut Eagle ( *S. isidori* ), Rufous‐tailed Hawk ( *B. ventralis* ), Plumbeous Hawk ( *C. plumbea* ), Solitary Eagle ( *B. solitarius* ) and Chaco Eagle ( *B. coronatus* ), based on a maximum likelihood tree of mitogenomic sequences.

Phylogenetic relationships among raptor species were inferred using the complete 13 PCGs and 2 rRNA sequences. Most nodes in the resulting maximum‐likelihood tree were strongly supported, with bootstrap values exceeding 95% (Figure 3). Resolution was generally high across Accipitridae and Falconidae. Within Falconidae, caracaras (Phalcoboenus and Caracara) formed a clade distinct from true falcons (including *F. deiroleucus* ). Within Accipitridae, *M. guianensis* and *H. harpyja* were recovered as sister taxa with strong support, and *Spizaetus* species formed a well‐supported monophyletic clade within Aquilinae (booted or true eagles). *B. ventralis* clustered within the *Buteo* lineage with high bootstrap support, while *C. plumbea* was placed as sister to *Buteogallus* species (Figure 3).

**FIGURE 4:**
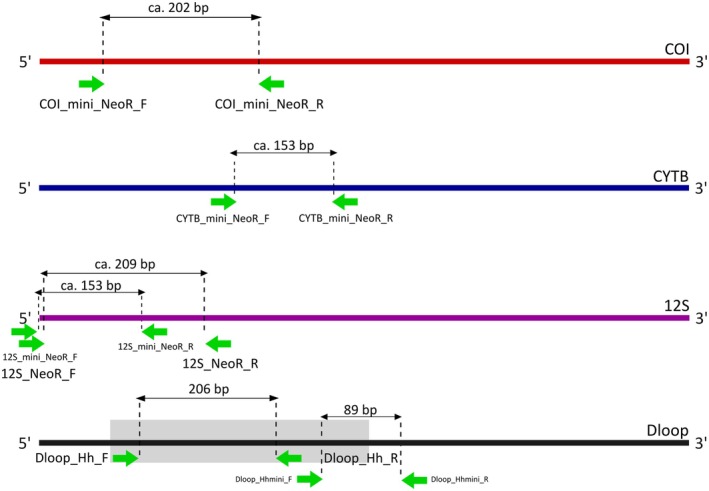
Relative positions of primer binding sites across the complete mitochondrial genes and the Control Region (the gray box indicates the characterized polymorphic region in *H. harpyja* ). The average total length of each barcode region is shown.

Using the assembled mitogenomes and reference sequences from Neotropical raptors, we designed in silico primers for metabarcoding applications targeting COI, CytB, 12S rRNA, and the Control Region (Table 1, Figure 4). Both COI and CytB proved suboptimal for metabarcoding due to high variability leading to either long fragments incompatible with degraded eDNA or short fragments lacking conserved primer‐binding sites. Primer design for these loci required numerous degenerate bases (Table 1), increasing the risk of non‐specific amplification (additional results in Table S4).

**TABLE 1:** Best primer pairs for targeted barcode amplification across mitochondrial markers in keystone Neotropical raptor species.

Region	Length	Name_F	Sequence_F 5′ → 3′	Tm	Name_R	Sequence_R 5′ → 3′	Tm
COI	~202	COI_mini_NeoR_F	NCGYATAAAYAAYATRAG	53.6	COI_mini_NeoR_R	NGGDGGYTTTATGTTRAT	56.8
12 s	~209	12S_NeoR_F	AGACTTAGTCCTAACCTT	55.0	12S_NeoR_R	ACAAGATTTACCARCCCT	57.0
12 s	~153	12S_mini_NeoR_F	AAAGACTTAGTCCTAACC	53.5	12S_mini_NeoR_R	AATGTTARTTACTGCTGA	51.8
CYTB	~153	CYTB_mini_NeoR_F	MYCAYACATGYCGAAAYG	64.5	CYTB_mini_NeoR_R	CHACRAAGGCDGTTGCTA	59.5
Dloop	~206	Dloop_NeoR_F	ATTCATATATATGTAATACGGGC	57.0	Dloop_NeoR_R	AGATAACCTGGTCCGACA	60.2
Dloop	~89	Dloop_mini_NeoR_F	GCACTTCTTGCTCTTTTGC	60.6	Dloop_mini_NeoR_R	GCCGCGATTAAGAGGCA	62.9

Our most effective barcode candidate was the 12S ribosomal RNA gene. The longer (~209 bp) version exhibited highly conserved primer‐binding regions (Table 1, Figure 4), identifying all target species of conservation concern. This marker achieved an overall ~95% discrimination power, failing only to distinguish between the allopatric pair *Pseudastur albicollis* and *Pseudastur polionotus*. Our mini‐barcode version spanning ~153 bp required only one degenerate base in the reverse primer (Table 1), showing a similar resolution, but with an additional ambiguity between *Buteogallus aequinoctialis* and *Buteogallus anthracinus* , which are also largely allopatric. For *H. harpyja* , species‐specific primers targeting the Control Region were developed. The longer fragment (206 bp) resolved 20 of the 22 previously described haplotypes, but failed to differentiate between haplotypes 3 and 20, as the diagnostic polymorphism lies just beyond the 3′ end of the amplified region (at the extreme end of Domain I). Despite this limitation, the two haplotypes seem to occur in distinct geographic regions: haplotype 3 was found in Peru, while haplotype 20 was found in northern South America and Central America. To overcome this limitation and enable identification of all 22 haplotypes using shorter fragments compatible with eDNA metabarcoding approaches, we designed a mini‐barcode primer set (89 bp). These primers target regions flanking the key diagnostic polymorphism, allowing for full resolution of all haplotypes sourced from degraded DNA samples (Table 1, Figure 4).

## 4 Discussion

Neotropical raptors face significant conservation challenges, with most species either threatened or lacking adequate population data. In this study, we present the complete mitochondrial genome assemblies and annotations for nine emblematic raptor species of the Neotropics, including four threatened species: the Chaco Eagle ( *B. coronatus* ), Black‐and‐chestnut Eagle ( *S. isidori* ), Harpy Eagle ( *H. harpyja* ), and Rufous‐tailed Hawk ( *B. ventralis* ). We also included five near‐threatened and data‐deficient taxa: the Orange‐breasted Falcon ( *F. deiroleucus* ), Solitary Eagle ( *B. solitarius* ), Plumbeous Hawk ( *C. plumbea* ), Ornate Hawk‐Eagle ( *S. ornatus* ), and Crested Eagle ( *M. guianensis* ). In addition, we assessed the phylogenetic resolution of mitogenome sequences and developed in silico metabarcode primers for environmental DNA applications, thereby providing crucial resources for conservation genetics and biodiversity monitoring.

The overall mitochondrial genome structure observed across species was consistent with known avian patterns, including a conserved gene complement and a slight A + T bias (De Panis et al. 2021). Unlike the typical gene order seen in species such as *Gallus gallus* or New World vultures where the Control Region is located between tRNA‐Glu and tRNA‐Phe, the CR of the newly characterized raptor species is positioned between tRNA‐Thr and tRNA‐Pro. This is consistent with the mitochondrial architecture observed in many Falconidae and Accipitridae species such as *Falco peregrinus* (Mindell et al. 1998), *Buteo buteo* (Haring et al. 2001) and Old World vultures (Li et al. 2015; Mereu et al. 2017). However, while our assembly and annotation pipeline successfully recovered complete mitochondrial genomes with all Control regions matching expected references, some technical limitations may remain. For instance, short‐read Illumina sequencing may introduce uncertainties in highly repetitive regions of the mitochondrial genome, such as the control region. While these limitations should not impact the recovery of coding regions, they can obscure the precise characterization of tandem repeats, thereby affecting total length estimates. Thus, experimental validation using complementary approaches (e.g., long‐read sequencing or targeted PCR confirmation) would further refine structural features and strengthen future analyses of repetitive regions.

To test the phylogenetic resolution of the assembled mitogenomes, we analyzed the systematics of taxonomically challenging groups such as Accipitridae, which encompasses several cosmopolitan species of hawks, Old World vultures and eagles. Overall, our phylogenetic reconstruction of raptor species was supported by high bootstrap values across most nodes. An exception to this pattern was observed in the Cathartidae family, which, although not a focal group in our study, was included in the analysis and exhibited limited phylogenetic resolution, with a low bootstrap value of 50 for the sister relationship between *Sarcoramphus* and *Gymnogyps*. This group has previously been described as challenging to resolve using a combination of nuclear and mitochondrial loci (Johnson et al. 2016). Nonetheless, recent phylogenetic analyses using ultraconserved elements (UCEs) recovered *Sarcoramphus* and *Vultur* as sister taxa with strong support (Catanach et al. 2025). Within falcons, our analysis confirmed the traditional classification placing caracaras (*Phalcoboenus* and *Caracara*) in a distinct clade from true falcons (Fuchs et al. 2015). Both *Morphnus* and *Harpia* were placed as sister taxa, consistent with the traditional subfamily Harpiinae (*Harpia*, *Morphnus*, *Harpyopsis* and *Macheiramphus*), showing a similar branching order described in previous phylogenies (Lerner and Mindell 2005; Mindell et al. 2018), which place the subfamily as sister to Aquilinae (eagle species, including our target *Spizaetus* spp.). However, this branching pattern differs from recent UCE‐based reconstruction showing Harpiinae as a sister group to Buteoninae + Accipitrinae (Catanach et al. 2025). Finally, our phylogenomic analysis recovered the subfamily Buteoninae composed of two major subclades corresponding to the tribes Milvini (*Haliaeetus*, *Milvus*, and *Haliastur*) and Buteonini (*Buteo*, *Cryptoleucopteryx*, *Buteogallus*). Yet, our analysis placed *Butastur* in Milvini instead of the traditionally Buteonini clade, likely due to incomplete taxon sampling, requiring further deepening and inclusion of other species (do Amaral et al. 2009; Mindell et al. 2018; Catanach et al. 2025). Furthermore, although mitochondrial genomes provide strong resolution for species‐level phylogenetics and identification, mtDNA alone does not capture the full spectrum of genetic diversity. Thus, nuclear genomic markers will be essential to complement these data and resolve complex phylogenetic relationships and provide insight into genetic structure, hybridization, and adaptive evolution.

Beyond their phylogenetic utility, our comparative evaluation of mitochondrial loci revealed marked differences in their suitability for metabarcoding applications. We first focused on the standard barcode for animal species, the Cytochrome *c* Oxidase subunit I (Ratnasingham and Hebert 2007), and also evaluated the Cytochrome *b*, a traditional marker for raptor phylogenetics (e.g., Seibold and Helbig 1995; Wink 1995; Wink et al. 1998), which has proven effective for species identification in some taxa (Deiner et al. 2017; Ruppert et al. 2019). Our best candidate barcodes for both loci with a reasonable fragment size (< ~200 bp) achieved a high discrimination power, failing only to distinguish between *Buteo ventralis* and *Buteo jamaicensis*. However, as these species are allopatric, geographic separation minimizes the risk of misidentification. Although COI and CytB achieved high discrimination power, their high sequence variability required extensive primer degeneracy, increasing the risk of non‐specific amplification and limiting their utility for degraded environmental DNA (Deagle et al. 2014). Our most effective barcode candidate was the 12S ribosomal RNA gene, which is well‐suited for eDNA studies using degraded samples. Our mini‐barcode version is further expected to enhance species detection in samples containing trace or highly degraded DNA, such as soil, decomposed remains, or even air samples (Epp et al. 2012; Clare et al. 2022; Goray et al. 2024). The choice of the 12S rRNA gene aligns with its growing status as a standard marker for animal metabarcoding, due to the unique features of its RNA secondary structure (Riaz et al. 2011; Clarke et al. 2014; Deagle et al. 2014). This gene alternates between conserved and variable regions, with stable stem structures providing consistent primer‐binding sites flanked by hypervariable loops that facilitate species‐level discrimination (Kocher et al. 2017).

The generation of complete mitochondrial genomes and the development of species‐specific eDNA markers provide critical genomic resources for evolutionary research and conservation of threatened Neotropical raptors. For instance, the Harpy Eagle is the focus of national conservation programs across much of its range, and recent assessments have also suggested the potential need for ex situ conservation and captive management strategies (Vargas et al. 2025; Boss et al. 2022; de Oliveira et al. 2022). However, previous genetic studies have demonstrated that the Harpy Eagle exhibits remarkably high mitochondrial diversity, with most haplotypes showing strong geographic differentiation both within South America and between Central and South America (Lerner et al. 2009), highlighting the importance of expanded genetic surveys to inform reintroduction efforts and breeding plans. Similarly, the Orange‐breasted Falcon has been the subject of intensive captive‐breeding and reintroduction programs in Central America (Berry et al. 2020). Despite these efforts, substantial uncertainty remains regarding its current distribution, indicating that additional surveys are still required, particularly in Honduras, Nicaragua, and Panama (Thorstrom et al. 2002; Berry et al. 2020).

In Argentina, conservation studies of the Chaco Eagle have suggested a limited genetic bottleneck, potentially buffered by connectivity among populations. Nevertheless, no clearly differentiated genetic populations have been identified to date, emphasizing the need for further sampling across the northern portion of the species' distribution in Paraguay, Bolivia, and Brazil (Canal et al. 2017). Finally, international collaborative initiatives aimed at conserving the Black‐and‐chestnut Eagle seek to preserve its wild populations in the face of persistent threats, particularly persecution (Rivas‐Fuenzalida, Grande, et al. 2024; Vargas et al. 2025). Addressing these pressures will require additional research to evaluate the effects of habitat loss on dispersal and the maintenance of population connectivity in highly fragmented ecosystems (Zuluaga et al. 2022). In this context, the integration of eDNA‐based approaches provides a powerful, non‐invasive framework to improve our understanding of biogeographic patterns and intraspecific genetic variation, potential gene flow corridors, and ultimately support a more informed delineation of conservation units and management strategies for these key raptor species.

In summary, these results provide the genomic baseline necessary to implement eDNA monitoring programs across the Neotropics. This approach is particularly useful for studying the evolutionary biogeography of elusive raptors in remote or logistically challenging terrain where traditional surveys are often impractical. Integrating these molecular tools into existing conservation initiatives will help to improve the accuracy of conservation status assessments and the efficacy of management plans for threatened Neotropical raptors.

## Author Contributions

**Diego De Panis:** data curation (supporting), investigation (supporting), methodology (supporting), project administration (equal). **Octavio Priotto:** investigation (supporting), resources (supporting), visualization (supporting). **Julián Padró:** conceptualization (lead), formal analysis (lead), investigation (lead), methodology (equal), project administration (equal).

## Conflicts of Interest

The authors declare no conflicts of interest.

## Supporting information


**Data S1:** ece373262‐sup‐0001‐Supinfo.pdf.

## Data Availability

All mitochondrial genome sequences generated in this study have been deposited in GenBank (see Supporting Information for accession numbers).
